# Changing Age Distribution of the COVID-19 Pandemic — United States, May–August 2020

**DOI:** 10.15585/mmwr.mm6939e1

**Published:** 2020-10-02

**Authors:** Tegan K. Boehmer, Jourdan DeVies, Elise Caruso, Katharina L. van Santen, Shichao Tang, Carla L. Black, Kathleen P. Hartnett, Aaron Kite-Powell, Stephanie Dietz, Matthew Lozier, Adi V. Gundlapalli

**Affiliations:** ^1^CDC COVID-19 Response Team; ^2^ICF International Inc., Atlanta, Georgia.

As of September 21, 2020, the coronavirus disease 2019 (COVID-19) pandemic had resulted in more than 6,800,000 reported U.S. cases and more than 199,000 associated deaths.* Early in the pandemic, COVID-19 incidence was highest among older adults (*1*). CDC examined the changing age distribution of the COVID-19 pandemic in the United States during May–August by assessing three indicators: COVID-19–like illness-related emergency department (ED) visits, positive reverse transcription–polymerase chain reaction (RT-PCR) test results for SARS-CoV-2, the virus that causes COVID-19, and confirmed COVID-19 cases. Nationwide, the median age of COVID-19 cases declined from 46 years in May to 37 years in July and 38 in August. Similar patterns were seen for COVID-19–like illness-related ED visits and positive SARS-CoV-2 RT-PCR test results in all U.S. Census regions. During June–August, COVID-19 incidence was highest in persons aged 20–29 years, who accounted for >20% of all confirmed cases. The southern United States experienced regional outbreaks of COVID-19 in June. In these regions, increases in the percentage of positive SARS-CoV-2 test results among adults aged 20–39 years preceded increases among adults aged ≥60 years by an average of 8.7 days (range = 4–15 days), suggesting that younger adults likely contributed to community transmission of COVID-19. Given the role of asymptomatic and presymptomatic transmission (*2*), strict adherence to community mitigation strategies and personal preventive behaviors by younger adults is needed to help reduce their risk for infection and subsequent transmission of SARS-CoV-2 to persons at higher risk for severe illness.

CDC examined age trends during May–August for 50 states and the District of Columbia (DC) using three indicators: 1) COVID-19–like illness-related ED visits; 2) positive SARS-CoV-2 RT-PCR test results; and 3) confirmed COVID-19 cases. COVID-19–like illness-related ED visits, reported by health facilities to the National Syndromic Surveillance Program (NSSP),^†^ had fever with cough, shortness of breath, or difficulty breathing in the chief complaint text or a discharge diagnostic code for COVID-19 and no diagnostic codes for influenza.^§^ Analyses of COVID-19–like illness-related ED visits were based on the ED visit date.

SARS-CoV-2 RT-PCR test results were obtained from COVID-19 electronic laboratory reporting data submitted by state health departments (37 states) and, when age was unavailable in state-submitted data, from data submitted directly by public health, commercial, and reference laboratories (13 states and DC).^¶^ Data represent the number of specimens tested, not individual persons who received testing. Analyses were based on the specimen collection date or test order date.** The daily percentage of positive SARS-CoV-2 test results (percent positivity) was calculated as the number of positive test results divided by the sum of positive and negative test results.

Confirmed COVID-19 cases were identified from individual-level case reports submitted by state health departments^††^; analyses were based on the date the case was reported to CDC.^§§^ Confirmed COVID-19 cases had a positive SARS-CoV-2 RT-PCR test result. Case data represent individual persons (some of whom might have had multiple positive test results). Monthly incidence was calculated using 2018 U.S. Census population estimates.

National case counts, percentage distributions, and estimated incidence of confirmed COVID-19 cases were calculated by 10-year age increments and by month (May–August). The weekly median age of persons with COVID-19–like illness-related ED visits, positive SARS-CoV-2 test results, and confirmed COVID-19 cases, as well as that of persons for whom all SARS-CoV-2 tests were conducted, were plotted nationally for the four U.S. Census regions. To minimize the impact of testing availability on findings, the early pandemic period (January–April) was excluded.

The southern United States experienced regional COVID-19 outbreaks during June–July 2020. For U.S. Department of Health and Human Services (HHS) Regions 4, 6, and 9,^¶¶^ daily percent positivity was plotted for four age groups (0–19 years, 20–39 years, 40–59 years, and ≥60 years). The segmented package (version 1.2-0) in R software (version 3.6.0; The R Foundation) was used to segment the age group-specific trend lines and identify inflection points when the slopes changed.

National incidence of confirmed COVID-19 increased from 185 cases per 100,000 persons in May to 316 in July, then declined to 275 in August (Table). During May–July, incidence increased among persons in all age groups <80 years, with the largest increases in persons aged <30 years. As a result, the median age of confirmed COVID-19 cases decreased from 46 years in May to 37 years in July and 38 years in August. During June–August, incidence was highest among persons aged 20–29 years, who accounted for the largest proportion of total cases (>20%). Similar age shifts were observed nationwide.

**TABLE:** Reported number of confirmed* COVID-19 cases and estimated incidence,^†^ by age group^§^ and month — United States, May 1–Aug 31, 2020

Age group (yrs)	May 2020		June 2020		July 2020			Aug 2020	
	No. (%)	Incidence^†^	No. (%)	Incidence^†^	No. (%)	Incidence^†^	No. (%)		Incidence^†^
0–9	13,987 (2.3)	35.0	24,772 (3.3)	61.9	40,093 (3.9)	100.2	35,612 (4.0)		89.0
10–19	31,053 (5.1)	74.0	55,596 (7.5)	132.4	104,048 (10.1)	247.9	103,637 (11.5)		246.9
20–29	93,741 (15.5)	206.3	149,761 (20.2)	329.6	240,105 (23.2)	528.5	189,366 (21.0)		416.8
30–39	101,917 (16.9)	233.2	130,415 (17.6)	298.4	183,478 (17.8)	419.9	148,500 (16.5)		339.8
40–49	98,982 (16.4)	244.6	119,043 (16.0)	294.2	157,019 (15.2)	388.1	134,288 (14.9)		331.9
50–59	99,058 (16.4)	231.3	108,509 (14.6)	253.4	139,004 (13.4)	324.6	124,835 (13.9)		291.5
60–69	72,115 (11.9)	192.7	73,225 (9.9)	195.7	89,586 (8.7)	239.4	84,247 (9.4)		225.1
70–79	42,476 (7.0)	187.3	40,714 (5.5)	179.6	47,851 (4.6)	211.1	47,060 (5.2)		207.6
≥80	51,241 (8.5)	404.4	41,023 (5.5)	323.7	32,370 (3.1)	255.4	33,005 (3.7)		260.5
**Total**	**604,570 (100.0)**	**184.8**	**743,058 (100.0)**	**227.1**	**1,033,554 (100.0)**	**315.9**	**900,550 (100.0)**		**275.3**

**Abbreviation:** COVID-19 = coronavirus disease 2019.

* A confirmed COVID-19 case required detection of SARS-CoV-2 RNA in a clinical specimen using a molecular amplification detection test. ^†^ Cases per 100,000 population calculated using 2018 U.S. Census population estimates.

^§^ Data from individual-level case reports submitted by state health departments, using date case was reported to CDC. Case report data were available for approximately 68% of the total aggregate counts of confirmed cases submitted by state health departments. Case reports with missing information on age (3,845) were not included.

The median age trend lines for all three indicators (COVID-19–like illness-related ED visits, positive SARS-CoV-2 test results, and confirmed COVID-19 cases) followed similar patterns in the national data (Figure 1) and within each U.S. Census region (Figure 2); however, patterns differed by region. Nationally and in the South and Midwest, median age decreased until mid- to late June, increased during July, and decreased in the latter half of August. In the West, median age declined from May to mid-June and then remained relatively stable or slightly increased during July–August. In the Northeast, median age of persons with positive test results and confirmed cases was stable in May, decreased sharply in June, increased slightly in July, and decreased in August; median age for persons with COVID-19–like illness-related ED visits declined steadily from mid-June to mid-August. In all four U.S. Census regions, the median age of persons for whom all SARS-CoV-2 tests were conducted was relatively stable in May (whereas median age of persons with positive test results and confirmed cases declined in May) and began to decrease following declines in the other three indicators.

**FIGURE 1:**
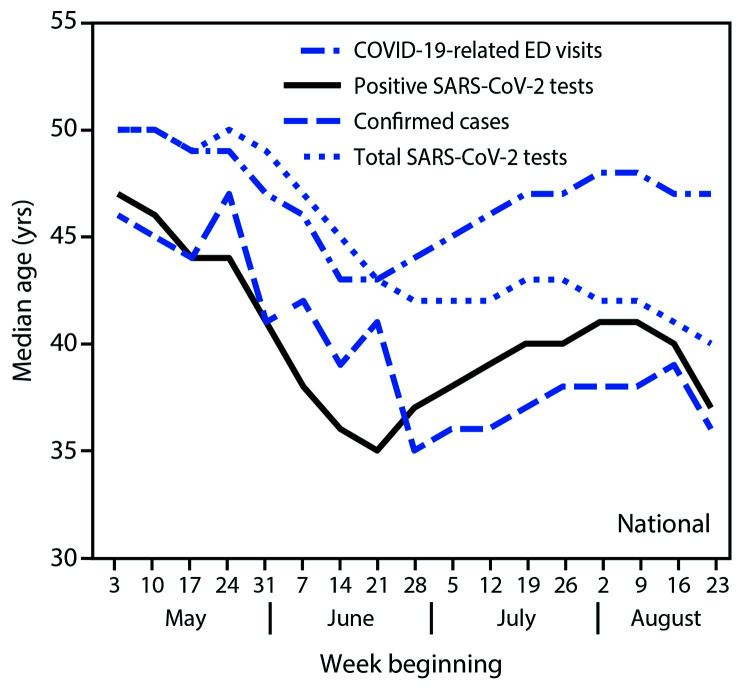
Weekly median age of persons with COVID-19–like illness-related emergency department (ED) visits,* positive SARS-CoV-2 reverse transcription–polymerase chain reaction (RT-PCR) test results,**^†^** and confirmed COVID-19 cases,**^§^** and of persons for whom all SARS-CoV-2 RT-PCR tests were conducted**^¶^** — United States, May 3–August 29, 2020 **Abbreviation:** COVID-19 = coronavirus disease 2019. * From CDC National Syndromic Surveillance Program (NSSP), using date of ED visit. NSSP records 73% of all emergency department visits in the United States. ^†^ From COVID-19 electronic laboratory reporting data submitted by state health departments for 37 states and from data submitted directly by public health, commercial, and reference laboratories for 13 states and the District of Columbia, based on specimen collection or test order date. The data might not include results from all testing sites within a jurisdiction (e.g., point-of-care test sites) and therefore reflect the majority of, but not all, SARS-CoV-2 RT-PCR tests in the United States. ^§^ From case reports with individual-level information submitted by state health departments, using date case was reported to CDC. Case report data were available for approximately 68% of the total daily aggregate number of confirmed cases submitted by state health departments. ^¶^ From COVID-19 electronic laboratory reporting data submitted by state health departments for 37 states and from data submitted directly by public health, commercial, and reference laboratories for 13 states and the District of Columbia, based on specimen collection or test order date. The data might not include results from all testing sites within a jurisdiction (e.g., point-of-care test sites) and therefore reflect the majority of, but not all, SARS-CoV-2 RT-PCR tests in the United States.

**FIGURE 2:**
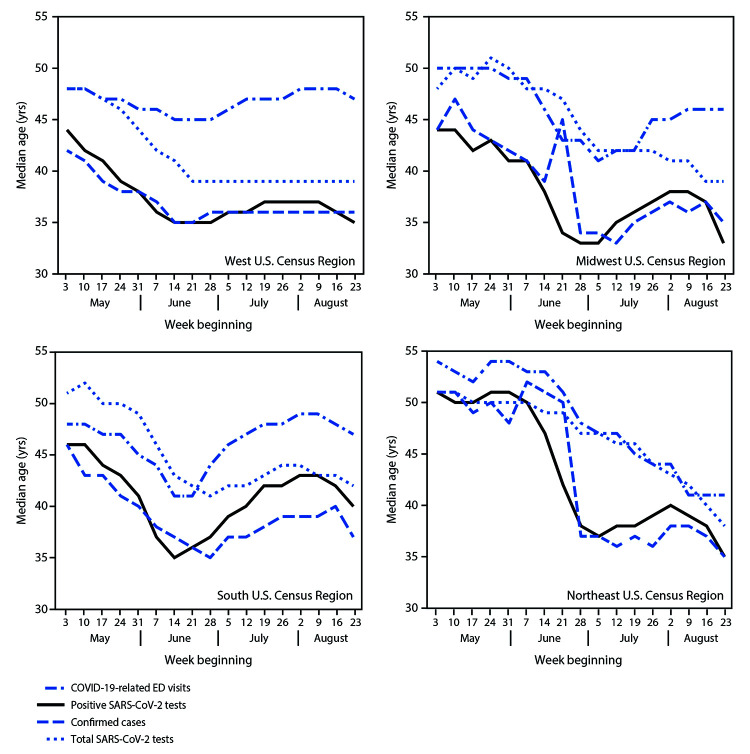
Weekly median age of persons with COVID-19–like illness-related emergency department (ED) visits,* positive SARS-CoV-2 reverse transcription–polymerase chain reaction (RT-PCR) test results,**^†^** and confirmed COVID-19 cases,**^§^** and of persons for whom all SARS-CoV-2 RT-PR tests were conducted,**^¶^** by U.S. Census region** — United States May 3–August 29, 2020 **Abbreviation:** COVID-19 = coronavirus disease 2019. * From CDC National Syndromic Surveillance Program (NSSP), using date of ED visit. NSSP records 73% of all emergency department visits in the United States. ^†^ From COVID-19 electronic laboratory reporting data submitted by state health departments for 37 states and from data submitted directly by public health, commercial, and reference laboratories for 13 states and the District of Columbia, based on specimen collection or test order date. The data might not include results from all testing sites within a jurisdiction (e.g., point-of-care test sites) and therefore reflect the majority, but not all, SARS-CoV-2 RT-PCR tests in the United States. ^§^ From case reports with individual-level information submitted by state health departments, using date case was reported to CDC. Case report data were available for approximately 68% of the total daily aggregate number of confirmed cases submitted by state health departments. ^¶^ From COVID-19 electronic laboratory reporting data submitted by state health departments for 37 states and from data submitted directly by public health, commercial, and reference laboratories for 13 states and the District of Columbia, based on specimen collection or test order date. The data might not include results from all testing sites within a jurisdiction (e.g., point-of-care test sites) and therefore reflect the majority, but not all, SARS-CoV-2 RT-PCR tests in the United States. ** *West*: Alaska, Arizona, California, Colorado, Hawaii, Idaho, Montana, Nevada, New Mexico, Oregon, Utah, Washington, and Wyoming; *Midwest*: Illinois, Indiana, Iowa, Kansas, Michigan, Minnesota, Missouri, Nebraska, North Dakota, Ohio, South Dakota, and Wisconsin; *South*: Alabama, Arkansas, Delaware, District of Columbia, Florida, Georgia, Kentucky, Louisiana, Maryland, Mississippi, North Carolina, Oklahoma, South Carolina, Tennessee, Texas, Virginia, and West Virginia; *Northeast*: Connecticut, Maine, Massachusetts, New Hampshire, New Jersey, New York, Pennsylvania, Rhode Island, and Vermont.

During June 2020 in HHS Regions 4, 6, and 9, the change to an upward slope in percent positivity among persons aged 20–39 years occurred an average of 8.7 days (range 4–15 days) before the change to an upward slope among persons aged ≥60 years (Supplementary Figure, https://stacks.cdc.gov/view/cdc/93914). This pattern was most evident in Region 4 (Southeast) where the increase in percent positivity among persons aged 20–39 years preceded increases among persons aged 40–59 years by 9 days and those aged ≥60 years by 15 days; percent positivity among persons aged 0–19 years increased steadily from early May to early July. Within HHS Regions 6 and 9 (Southcentral and Southwest), the percent positivity among persons aged 0–19, 20–39, and 40–59 years increased at approximately the same time and preceded increases among persons aged ≥60 years by approximately 7 days in Region 6 and 4 days in Region 9.

## Discussion

During June–August, the COVID-19 pandemic in the United States affected a larger proportion of younger persons than during January–May 2020 (*1*). The shift toward younger ages occurred in all four U.S. Census regions, regardless of changes in incidence during this period, and was reflected in COVID-19–like illness-related ED visits, positive SARS-CoV-2 RT-PCR test results, and confirmed COVID-19 cases. A similar age shift occurred in Europe, where the median age of COVID-19 cases declined from 54 years during January–May to 39 years during June–July, during which time persons aged 20–29 years constituted the largest proportion of cases (19.5%) (*3*).

Case and laboratory surveillance are based on consistent availability of diagnostic testing to all segments of the population, and changes in testing across age groups could affect the age distribution of positive SARS-CoV-2 test results and confirmed cases. Although testing availability has varied by place, time, and test provider, it is unlikely that the observed age shift resulted solely from changes in testing availability. First, the decline in median age of persons for whom all SARS-CoV-2 tests were conducted lagged behind declines in median age of persons with positive test results and confirmed cases, suggesting that infection patterns drove testing patterns. Second, the age distribution of persons for whom all SARS-CoV-2 tests were conducted shifted toward younger groups from May to June but remained relatively consistent during June–August. Third, the percent positivity continued to increase in the face of increased testing volume; this was most evident in HHS Regions 4 and 6 among persons aged 20–39 years during early to mid-June (Supplementary Figure, https://stacks.cdc.gov/view/cdc/93914). Fourth, the median age of persons with COVID-19–like illness-related ED visits, which is not dependent on testing availability, showed similar patterns to those of persons with positive test results and confirmed cases.

This report provides preliminary evidence that younger adults contributed to community transmission of COVID-19 to older adults. Across the southern United States in June 2020, the increase in SARS-CoV-2 infection among younger adults preceded the increase among older adults by 4–15 days (or approximately one to three incubation periods). Similar observations have been reported by the World Health Organization.*** Further investigation of community transmission dynamics across age groups to identify factors that might be driving infection among younger adults and subsequent transmission to older adults is warranted.

These findings have important clinical and public health implications. First, occupational and behavioral factors might put younger adults at higher risk for exposure to SARS-CoV-2. Younger adults make up a large proportion of workers in frontline occupations (e.g., retail stores, public transit, child care, and social services) and highly exposed industries (e.g., restaurants/bars, entertainment, and personal services) (*4*,*5*), where consistent implementation of prevention strategies might be difficult or not possible. In addition, younger adults might also be less likely to follow community mitigation strategies, such as social distancing and avoiding group gatherings (*6*,*7*). Second, younger adults, who are more likely to have mild or no symptoms,^†††^ can unknowingly contribute to presymptomatic or asymptomatic transmission to others (*2*), including to persons at higher risk for severe illness. Finally, SARS-CoV-2 infection is not benign in younger adults, especially among those with underlying medical conditions,^§§§^ who are at risk for hospitalization, severe illness, and death (*8*).

The findings in this report are subject to at least five limitations. First, case report data submitted to CDC by state health departments underestimates true incidence. Second, batch reporting of historical cases by some states might have led to spikes in median age trend lines, such as the increase seen in the Midwest region in June. Third, the report’s three data sources varied in their geographic coverage, with laboratory data being the most comprehensive. Nevertheless, consistent patterns and trends were observed across the three indicators. Fourth, analyzing data at a regional level could minimize differences in age group–specific trends that might otherwise be observed at the state or local level. Finally, use of ten- and twenty-year age groups might mask age patterns among smaller age groups and those that cross decades, such as recent increases in COVID-19 cases among college and university students.^¶¶¶^

Increased prevalence of SARS-CoV-2 infection among younger adults likely contributes to community transmission of COVID-19, including to persons at higher risk for severe illness, such as older adults. Emphasis should be placed on targeted mitigation strategies to reduce infection and transmission among younger adults, including age-appropriate prevention messages (*7*), restricting in-person gatherings and events,**** recommending mask use and social distancing in settings where persons socialize,^††††^ implementing safe practices at on-site eating and drinking venues (*9*), and enforcing protection measures for essential and service industry workers.^§§§§^ Given the role of asymptomatic and presymptomatic transmission (*2*), all persons, including young adults, should take extra precautions to avoid transmission to family and community members who are older or who have underlying medical conditions. Strict adherence to community mitigation strategies and personal preventive behaviors by younger adults is needed to help reduce their risk for infection and minimize subsequent transmission of SARS-CoV-2 to persons at higher risk for severe COVID-19.

**Summary**

**What is already known about this topic?**

Early in the pandemic, COVID-19 incidence was highest among older adults.

**What is added by this report?**

During June–August 2020, COVID-19 incidence was highest in persons aged 20–29 years, who accounted for >20% of all confirmed cases. Younger adults likely contribute to community transmission of COVID-19. Across the southern United States in June 2020, increases in percentage of positive SARS-CoV-2 test results among adults aged 20–39 years preceded increases among those aged ≥60 years by 4–15 days.

**What are the implications for public health practice?**

Strict adherence to community mitigation strategies and personal preventive behaviors by younger adults is needed to help reduce infection and subsequent transmission to persons at higher risk for severe illness.

## Footnotes

* https://www.cdc.gov/covid-data-tracker/index.html#trends.

† During May–August, an average of 3,679 facilities in 47 states and DC reported to the National Syndromic Surveillance Program representing 73% of total ED visits nationwide. Data from Hawaii, South Dakota, and Wyoming were not included. https://www.cdc.gov/nssp/participation-coverage-map.html.

§ The query for COVID-19–like illness is applied in the ESSENCE system and includes COVID-19 symptoms (fever AND either cough, OR difficulty breathing, OR shortness of breath) or coronavirus diagnostic codes. The query excludes diagnostic codes related to influenza.

¶ COVID-19 electronic laboratory reporting data submitted by state health departments from all laboratories performing SARS-CoV-2 RT-PCR testing were used for 37 states (Alabama, Alaska, Arizona, Arkansas, Colorado, Connecticut, Florida, Georgia, Hawaii, Idaho, Illinois, Indiana, Iowa, Kansas, Kentucky, Louisiana, Maryland, Massachusetts, Michigan, Minnesota, Montana, Nebraska, Nevada, New Hampshire, New Jersey, North Carolina, Oregon, Pennsylvania, South Carolina, South Dakota, Tennessee, Texas, Utah, Vermont, Virginia, West Virginia, and Wisconsin). SARS-CoV-2 RT-PCR testing data from a subset public health, commercial, and reference laboratories were used for DC and 13 states (California, Delaware, Maine, Mississippi, Missouri, New Mexico, New York, North Dakota, Ohio, Oklahoma, Rhode Island, Washington, and Wyoming). The data from the public health, commercial, and reference laboratories represent approximately 50% of all tests. The data might not include results from all testing sites within a jurisdiction (e.g., point-of-care test sites) and therefore reflect the majority, but not all, SARS-CoV-2 RT-PCR tests in the United States. The data represent laboratory test totals, not individual persons, and exclude antibody and antigen tests.

** Within COVID-19 electronic laboratory reporting data: state was assigned using location of the state health department that reported the test (available for 100% of tests), specimen collection date was used to assign date (available for approximately 98% of tests), and records with missing specimen collection data were excluded. Within data submitted directly by public health, commercial, and reference laboratories: state was assigned using patient location for 96% of tests, provider location was substituted for 1%, and records with both location fields missing (3%) were excluded; order date was used for 80% of tests, specimen collection date was substituted for 19%, and records with both date fields missing (1%) were excluded.

†† Throughout the COVID-19 pandemic, CDC has been tracking both aggregate and individual (i.e., line-list) counts of cases and deaths. CDC official counts of cases and deaths, released daily on https://www.cdc.gov/coronavirus/2019-ncov/cases-updates/cases-in-us.html, are aggregate counts from reporting jurisdictions. Some jurisdictions electronically submit standardized information for individual cases of COVID-19 to CDC using a case report form (https://www.cdc.gov/coronavirus/2019-ncov/php/reporting-pui.html) or the National Notifiable Diseases Surveillance System (NNDSS; https://wwwn.cdc.gov/nndss; https://wwwn.cdc.gov/nndss/covid-19-response.html). Individual-level case report data were available for approximately 68% of the aggregate number of confirmed cases.

§§ CDC report date, the date the case was reported to CDC by the state health department, was used because it is the most complete date variable across jurisdictions. If CDC report date was missing, report date was populated with the earliest date in a series of variables submitted by the jurisdiction, including hospital or ICU admission and discharge date, diagnosis date, symptom onset and resolution dates, and positive specimen dates. As of September 7, 2020, approximately 10% of reported COVID-19 confirmed cases in the 50 states and DC had no available date information; an estimation of how many of these were reported to CDC during May–August 2020 (the period of analysis for this study) is not possible at this time.

¶¶ HHS *Region 4:* Alabama, Florida, Georgia, Kentucky, Mississippi, North Carolina, South Carolina, and Tennessee; *Region 6:* Arkansas, Louisiana, New Mexico, Oklahoma, and Texas; *Region 9:* Arizona, California, Hawaii, and Nevada. https://www.hhs.gov/about/agencies/iea/regional-offices/index.html

*** https://www.who.int/westernpacific/news/speeches/detail/virtual-press-conference-on-covid-19-in-the-western-pacific.

††† https://arxiv.org/ftp/arxiv/papers/2006/2006.08471.pdf.

§§§ https://www.cdc.gov/coronavirus/2019-ncov/need-extra-precautions/people-with-medical-conditions.html.

¶¶¶ https://www.nytimes.com/interactive/2020/us/covid-college-cases-tracker.html.

**** https://www.cdc.gov/coronavirus/2019-ncov/community/large-events/considerations-for-events-gatherings.html.

†††† https://www.cdc.gov/coronavirus/2019-ncov/prevent-getting-sick/cloth-face-cover-guidance.html.

§§§§ https://www.cdc.gov/coronavirus/2019-ncov/community/organizations/businesses-employers.html.

## References

[1] Stokes EK, Zambrano LD, Anderson KN, Coronavirus disease 2019 case surveillance—United States, January 22–May 30, 2020. MMWR Morb Mortal Wkly Rep 2020;69:759–65. 10.15585/mmwr.mm6924e2 32555134 PMC7302472

[2] Furuse Y, Sando E, Tsuchiya N, Clusters of coronavirus disease in communities, Japan, January–April 2020. Emerg Infect Dis 2020;26:2176–9. 10.3201/eid2609.202272 32521222 PMC7454082

[3] European Centre for Disease Prevention and Control. Coronavirus disease 2019 (COVID-19) in the EU/EEA and the UK–eleventh update: resurgence of cases. Stockholm, Sweden: European Centre for Disease Prevention and Control; 2020. https://www.ecdc.europa.eu/sites/default/files/documents/covid-19-rapid-risk-assessment-20200810.pdf

[4] Rho HJ, Brown H, Fremstad S. A basic demographic profile of workers in frontline industries. Washington, DC: Center for Economic and Policy Research; 2020. https://cepr.net/wp-content/uploads/2020/04/2020-04-Frontline-Workers.pdf

[5] Dey M, Loewenstein MA, Piccone DS Jr, Polivka AE. Demographics, earnings, and family characteristics of workers in sectors initially affected by COVID-19 shutdowns. Washington, DC: US Department of Labor, Bureau of Labor Statistics; 2020. https://www.bls.gov/opub/mlr/2020/article/demographics-earnings-and-family-characteristics-of-workers-in-sectors-initially-affected-by-covid-19-shutdowns.htm

[6] Czeisler MÉ, Tynan MA, Howard ME, Public attitudes, behaviors, and beliefs related to COVID-19, stay-at-home orders, nonessential business closures, and public health guidance—United States, New York City, and Los Angeles, May 5–12, 2020. MMWR Morb Mortal Wkly Rep 2020;69:751–8. 10.15585/mmwr.mm6924e1 32555138 PMC7302477

[7] Nagata JM. Supporting young adults to rise to the challenge of COVID-19. J Adolesc Health 2020;67:297–8. 10.1016/j.jadohealth.2020.04.020 32405230 PMC7219379

[8] Cunningham JW, Vaduganathan M, Claggett BL, Clinical outcomes in young US adults hospitalized with COVID-19. JAMA Intern Med 2020. 10.1001/jamainternmed.2020.5313 32902580 PMC7489373

[9] Fisher KA, Tenforde MW, Feldstein LR, ; IVY Network Investigators; CDC COVID-19 Response Team. Community and close contact exposures associated with COVID-19 among symptomatic adults ≥18 years in 11 outpatient health care facilities—United States, July 2020. MMWR Morb Mortal Wkly Rep 2020;69:1258–64. 10.15585/mmwr.mm6936a5 32915165 PMC7499837

